# What kind of phonation causes the strongest vocal fold collision? – A hemi-larynx phonation contact pressure study

**DOI:** 10.1515/teme-2023-0002

**Published:** 2023-06-21

**Authors:** Florian Scheible, Raphael Lamprecht, Casey Schaan, Reinhard Veltrup, Marion Semmler, Alexander Sutor

**Affiliations:** Institute of Measurement and Sensor Technology, UMIT – Private University for Health Sciences, Medical Informatics and Technology, Hall in Tirol, Austria; Division of Phoniatrics and Pediatric Audiology, Department of Otorhinolaryngology, Head- and Neck Surgery, University Hospital Erlangen, Friedrich-Alexander-University Erlangen-Nürnberg, Erlangen, Germany

**Keywords:** contact-pressure, hemi-larynx ex vivo phonation experiment, thin film pressure sensor, vocal folds, Kontakt-Druck, dünner Foliendrucksensor, Stimmlippen, hemi-larynx ex vivo Phonationsexperiment

## Abstract

This paper presents a measurement setup which is able to measure the distribution of small scale pressure on an area of 15.2 mm × 30.4 mm with a sample rate up to 1.2 kHz. It was used to investigate the contact pressures of vocal folds during phonation. This was performed in ex vivo experiments of 11 porcine larynges. The contact pressure at the medial surface and other phonation parameters, as the glottal resistance and the closing velocity of the vocal fold, were measured at different adduction and elongation levels and air flow rates. A statistical analysis was carried out. It could be shown that the contact pressure rises, when the vocal fold is manipulated or when the flow rate is increased.

## Zusammenfassung

In diesem Artikel wird ein Messaufbau vorgestellt, mit dem die Verteilung von geringen Drücken auf einer Fläche von 15.2 mm × 30.4 mm mit einer Abtastrate von bis zu 1.2 kHz gemessen werden kann. Dieser Aufbau wurde verwendet, um die Kontaktdrücke der Stimmlippen während der Phonation zu untersuchen. Dazu wurden 11 Schweinekehlköpfe in ex vivo Experimenten untersucht. Die Kontaktdrücke entlang der medialen Stimmlippenoberfläche und andere Phonationsparameter wie der glottale Widerstand und die Geschwindigkeit beim Schließen der Stimmlippe wurden für verschiedene Adduktions- und Elongationsstufen sowie Luftflussraten gemessen. Es wurde eine statistische Analyse durchgeführt. Dadurch konnte statistisch signifikant gezeigt werden, dass der Kontaktdruck ansteigt, wenn die Stimmlippe manipuliert wird oder wenn die Flussrate erhöht wird.

## 1 Introduction

The spoken language is a fast and efficient way of communication, but many suffer from voice disorders [1]. The primary sound of the human voice is produced by the vocal folds (VFs) inside the larynx [2]. Intensive use of the voice can result in high impact forces between the VFs, this increases the chance of injuries of the VF tissue [3]. To understand the process of speaking it is important to study material properties of the tissue itself, which is done by different approaches like optical coherence tomography [4], ultrasound elastography [5, 6], optical measurement techniques [7–9] or pipette aspiration [10]. But beside of that, it is also of high interest to understand the interplay between the VFs and the air pressure from the lungs. During speech the phonation is changed through muscles acting in and on the larynx. The main manipulations are, on the one hand, elongation, which stretches the VFs, resulting in an increase of the fundamental frequency of vibration. On the other hand, adduction forces the VFs to close the glottis and press against each other [11]. Both manipulations are used in voice production to change acoustic parameters rapidly [12]. Another dominant role of our speech production plays the loudness of the voice, which is controlled by the laryngeal muscles but also by the airflow through the VFs. It can be distinguished between different types of phonation; mostly this is differentiated acoustically but it can also be done by some phonation parameters [13].

The contact pressure between phonating VFs was investigated previously on synthetic VFs by using a miniature pressure probe [14] or in hemi-larynx experiments [15]. *In vivo* measurements are mainly done by image-based approaches, where the contact pressure is estimated indirectly by videoendoscopy [16] or color doppler ultrasound [7]. Other *in vivo* approaches utilize a piezoresistive transducer [17] or a piezoelectric film [3].

The main challenges in measuring the contact pressure are the difficult accessibility, the small dimension, the low contact pressures, the variations of boundary conditions during the measurement process and the fast movement of the VFs.

We have developed a pressure sensing matrix readout unit, which is easily integrated to hemi-larynx experiments and is capable to measure the pressure two-dimensionally. In our series of hemi-larynx experiments with variations in VF manipulation, we hope to shed some light on understanding the variation in contact pressure between VFs during different types of phonation.

## 2 Methods

### 2.1 Sensor and read out unit

A commercial pressure mapping sensor (4201, Tekscan Inc., South Boston, MA, USA) with a piezoresistive sensing area of 21.1 × 45.7 mm^2^ and a density of 27.6 sensels cm^−2^ is chosen to measure the occurring pressure during phonation [18]. The manufacturer specifies the measurable pressure range up to 34 kPa. In order to fulfil the needs for the application, a custom read out unit was developed and a circuit board was designed [19].

**Figure 1:**
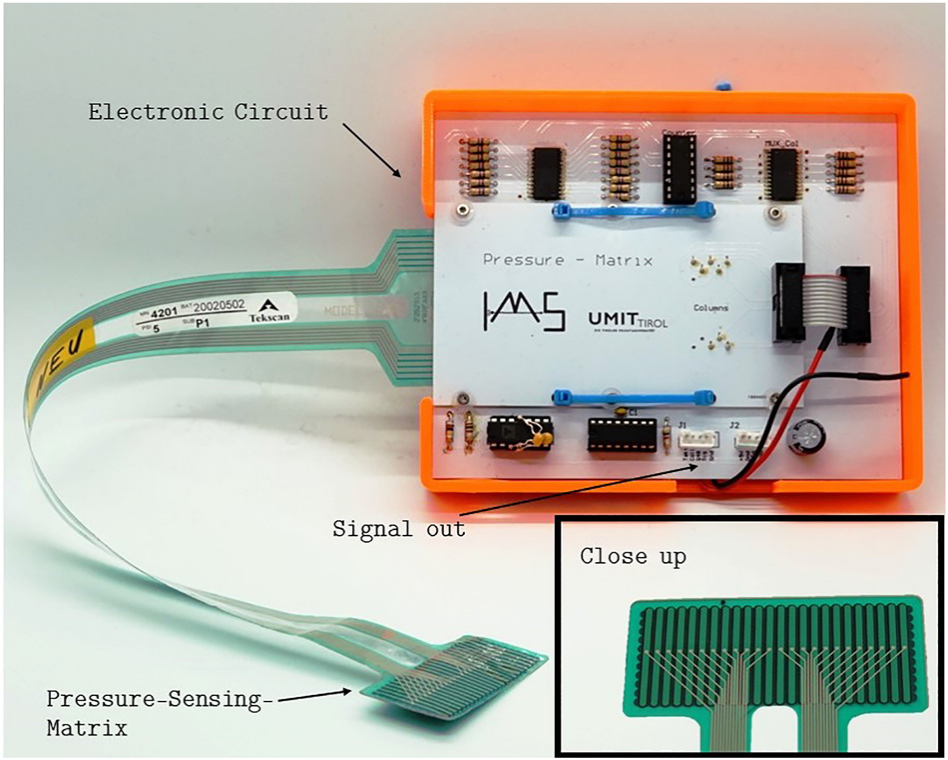
Pressure sensing matrix and the custom made read out unit.

The read out unit can be seen in Figure 1. It is based on two multiplexers switching between a reduced number of sensels, so the measured area was reduced to 15.2 mm × 30.4 mm and the sample rate could be increased. The setup is described in detail by Scheible et al. [19]. It was calibrated to pressures up to 17.6 kPa with a sample rate up to 1.2 kHz.

### 2.2 Anatomy & physiology of the larynx and vocal folds

The VFs are located inside the larynx and have a length of 10 mm–20 mm in anterior-posterior direction and a width of 8 mm–12 mm in medial-lateral direction [20]. The air from the lungs which flows through the VFs, forces them to vibrate. This vibration, also known as phonation, produces a pressure signal which is the sound source of the human voice. The main frequency of the vibration is called the fundamental frequency *f*_0_. To change the sound, this vibration can be manipulated with laryngeal muscles.

In the human speech different types of phonation exist, Sundberg [13] distinguished between them with the help of the glottal resistance, where a low glottal resistance indicates a “breathy” and a high glottal resistance a “pressed” voice. Further a “breathy” voice is indicated by the failure of the VFs to make contact. Therefore a “breathy” phonation will not be measured in this investigation. So when the denominations are used it should be understood as a phonation trending towards one of those types.

### 2.3 Larynx preparation

At the local slaughterhouse eleven larynges were gathered and immediately quick-frozen with 2-methylbutane (−150 °C). Shortly before the measurement they were unfrozen at 4 °C and later put in a NaCl solution for 15 min at room temperature. Superfluous tissue and the vestibular folds were removed to enable a straight view on the VF during phonation. In order to place the contact plate with the sensor along the original glottal midline the left VF was removed. The preparation was done according to Döllinger et al. [21].

The plate and the larynx were placed in the holder and mounted by screws to secure its position. The remaining holes and gaps between larynx and plate, where the air from the sub-glottal space could escape, is closed with protefix, an adhesive paste for dentures prosthesis.

**Figure 2:**
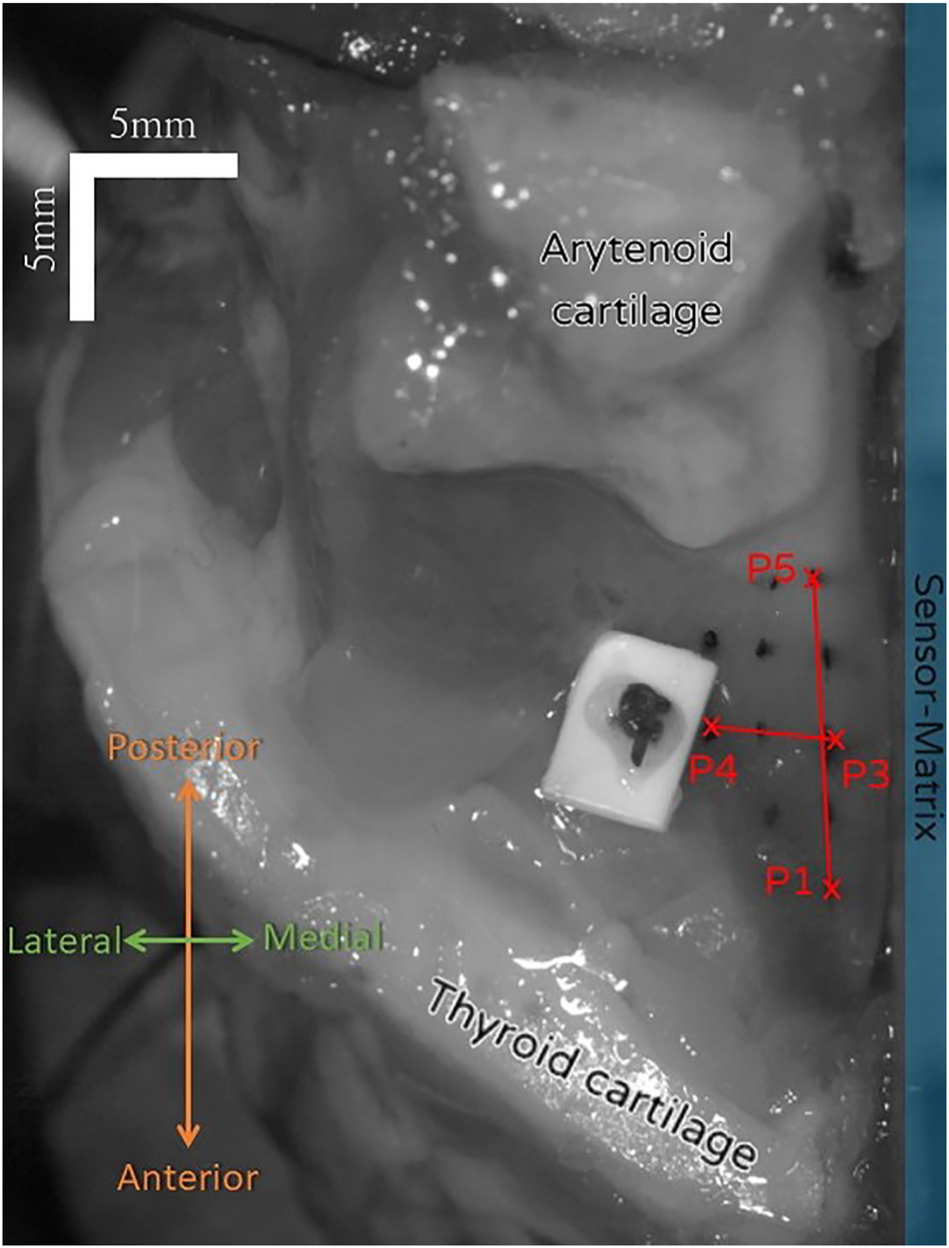
Snapshot of the highspeed camera. The marker points can be seen. On the right side the pressure sensor matrix is illustrated.

Figures 2 and 6 show the larynx mounted in the holder. If the vestibular folds are removed, the quality of the phonation suffers due to the lost weight of the removed tissue. In previous phonation studies a stabilization weight of 10 g is added and could produce good results [22].

### 2.4 Image recognition

Subsequently to the larynx preparation the VF is tattooed with marker points. In Figure 2 the inked markers as well as the measured length $l=\bar{P_{1}P_{5}}$ and width $w=\bar{P_{3}P_{4}}$ are indicated. The positions of the marker points are analyzed manually by selecting the marker position using a custom software.

### 2.5 Experimental setup

The setup is similar to the one described in a publication of our group where the sensor was already integrated in a hemi-larynx phonation experiment [19]. The prepared larynx is positioned in the holder which sits over the artificial trachea, where the remaining part of the porcine trachea is connected to. On the side of the removed VF the sensor, mounted on a plate, was positioned with a slight contact to the remaining VF. This can be seen in Figure 2. A mass flow controller then regulates the flow rate through the larynx. In order to prevent the biological tissue from drying out a humidifier was installed. The onset flow rate was found by increasing the flow until a clear sustained phonation was audible.

**Figure 3:**
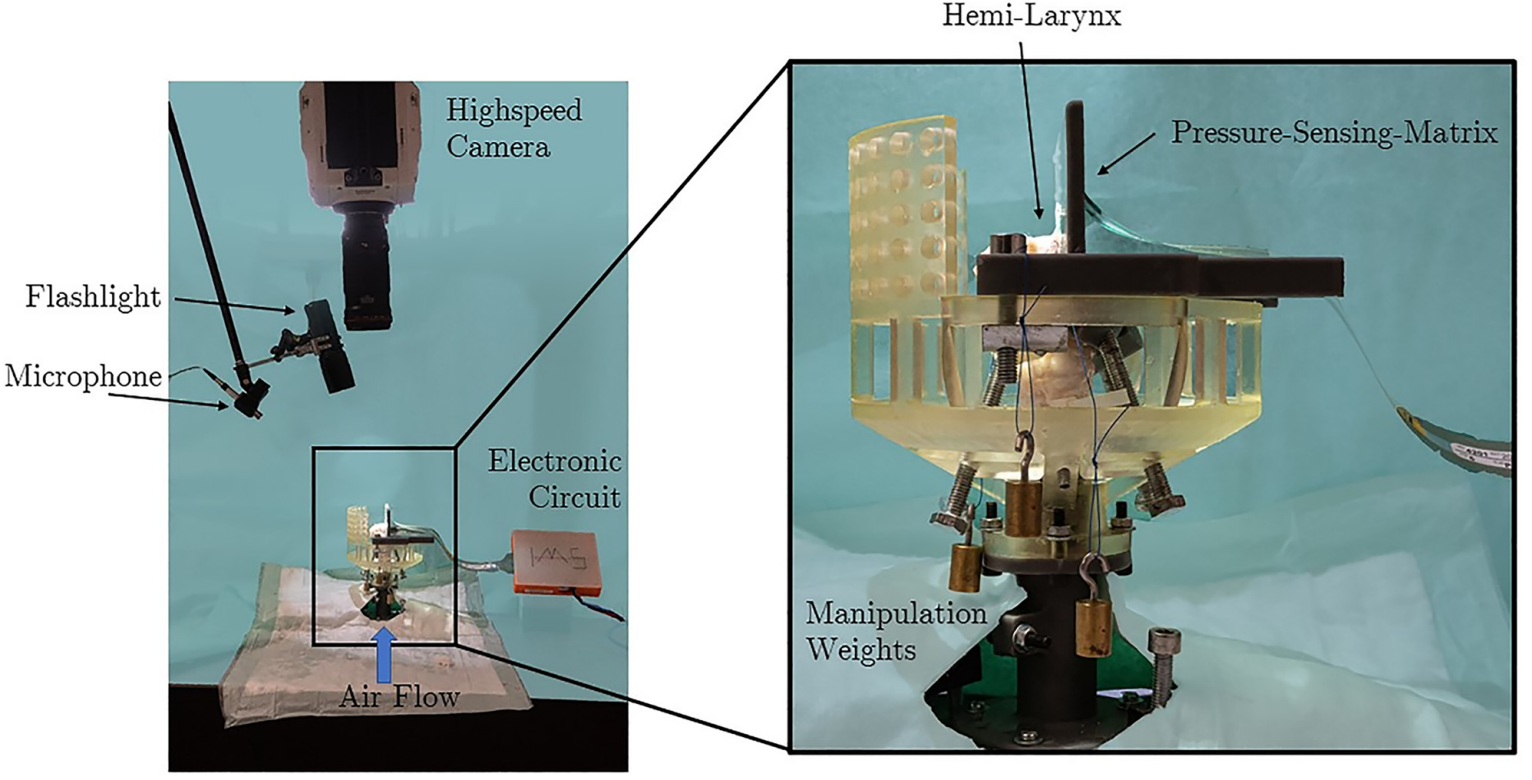
The experimental setup: the hemi-larynx is mounted on the artificial trachea inside the holder. The medial surface of the VF is oscillating against the pressure-sensing matrix mounted on a planar surface. From above, the high-speed camera records the movement and the microphone the sound of the vibrating VF.

During the measurement series with four equidistant flow steps the sub-glottal pressure, the audio signal (96 kS/s) as well as the contact pressure (1.2 kS/s) were recorded with National Instruments-measurement cards. The movement of the VF was recorded with a high-speed camera (4 kS/s) from above. All flow rates were maintained for 5 s. After a settle-time the recording was started for 0.5 s. The setup is shown in Figure 3.

### 2.6 Manipulations

During speech, the VFs undergo continuous manipulations. Important manipulations are adduction and elongation [11]. In a living species, these manipulations are performed by laryngeal muscles. An adduction is managed by an outer rotation of the arytenoid cartilage forced by the lateral cricoarytenoid muscle. An elongation is caused by a forward rotation of the thyroid cartilage caused by the contraction of the cricothyroid muscle [11]. To mimic these muscle activities, threads have been stitched in the arytenoid cartilage and thyroid cartilage. On the end of those threads weights apply force in order to adduct or elongate the VF, similar as it was done in previous studies [22].

**Figure 4:**
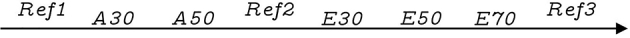
Cyclic order of manipulations.

Every larynx is manipulated in the same order, see Figure 4. It starts with a reference measurement Ref1, followed by adductions with 30 g (*A30*) and 50 g weights (*A50*). Then a second reference measurement Ref2 is made, before the larynx is elongated with 30 g (*E30*), 50 g (*E50*) and 70 g (*E70*). Lastly a third reference measurement Ref3 is made, this enables an observation of a potential hysteresis. All of those reference measurements were made with 10 g adduction.

### 2.7 Data

#### 2.7.1 Max. contact pressure at 50 % glottal line

Every larynx has a different size, therefore the position in relation to the sensor is documented by the camera from a vantage point above. The corresponding sensels to the 50 % glottal midline are then observed visually.

The maximal contact pressure is calculated as a difference between the highest and the lowest value of the contact pressure signal peaks, by doing so a potential air pressure is taken into account and subtracted.

#### 2.7.2 Phonation parameters

The high-speed camera recording of the phonation was analyzed with an in-house developed software tool (Glottis Analysis Tools 2020, Erlangen, Germany) [23]. This tool registers the contour of the open glottis and calculates the pixels distance to the glottal midline. So the distance between the VF and the glottal midline at 50 % in anterior-posterior direction is calculated, the so called glottal amplitude *GA*. With the help of that tool and other sensor data following parameters are determined:

– The fundamental frequency *f*_0_ is determined through a spectral analysis of the subglottal pressure. The peak with the lowest frequency value relates to the fundamental frequency *f*_0_.
– The instantaneous value of glottal velocity *v* calculated by the Glottis Analysis Tool by deriving the glottal amplitude *GA*,
(1) $$v=\frac{\partial }{\partial t}GA.$$
– The glottal resistance *RB* is an indicator for the power transfer between the glottal flow into the movement of the VF, the phonation efficiency so to say. It is an experimental parameter and is calculated of the ratio of the sub-glottal pressure *p*_sub_ to the given flow-rate *Q* by
(2) $$RB=\frac{\bar{p_{\text{sub}}}}{\bar{Q}}.$$

During the time of one phonation sequence the mean of both parameters was calculated [24, 25].

## 3 Results

### 3.1 Exemplary data of a single measurement

**Figure 5:**
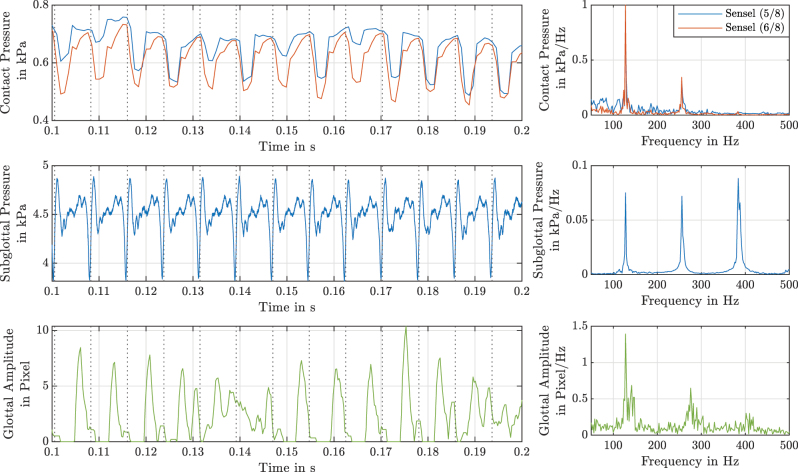
In the upper plot, the contact pressure of two sensels are drawn over a time range of 0.1 s, the middle one shows the subglottal pressure and in the lower one the glottal amplitude is plotted. Black dashed vertical lines mark the closing of the VF which is indicated by a sudden pressure drop followed by a quick rise. As one can observe it is also the moment of the contact pressure peak. On the right side the frequency domain of the whole measurement duration of 0.5 s are shown.

**Figure 6:**
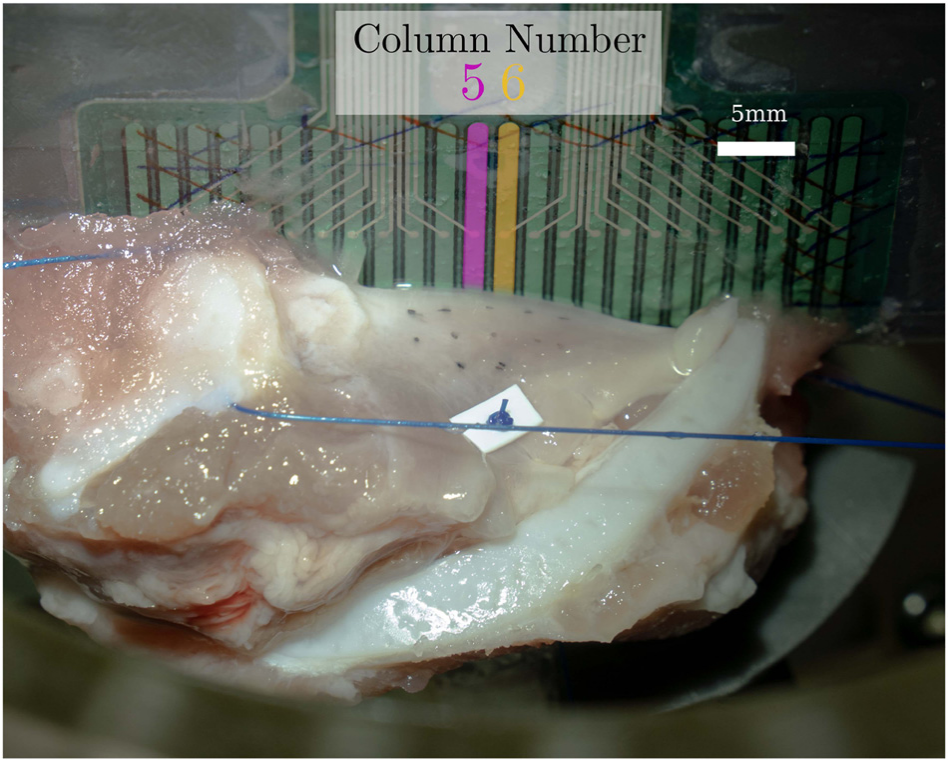
Two sensels of the eighth row, one in the fifth column (5/8) and next to it in the sixth (6/8) are selected for further demonstration. The eighth row is the bottom most and is hidden in behind the VF.

The contact pressures of two sensels (compare Figure 6), the sub-glottal pressure and the glottal amplitude are plotted in Figure 5. The moment of a complete glottis closure is indicated by a sudden pressure drop followed by a quick rise as can be seen in the sub-glottal pressure data. These instances are illustrated with dashed lines. All frequency domain data show peaks at 128 Hz, where the sub-glottal pressure and the glottal amplitude also shows higher spectral components, which are multiples of 128 Hz. These data were already shown in a former publication by our group [19]. Regarding the contact pressure wave forms, it can be seen, that a first plateau is followed by a high contact peak, subsequently the VF opens again. Similar curves can be found in literature gathered by measurements but also simulation [11, 15, 26].

### 3.2 Statistical analysis

Out of eleven prepared larynges only seven delivered data for all measurements and could be used for evaluation. The others did not show a clear phonation or a phonation was not possible within all manipulations.

The larynges did have a different onset flowrate, due to their size differences, the mean value of the onset flow rate was 15.4 slm (standard liter per minute).

#### 3.2.1 Deformation of the VF

**Figure 7:**
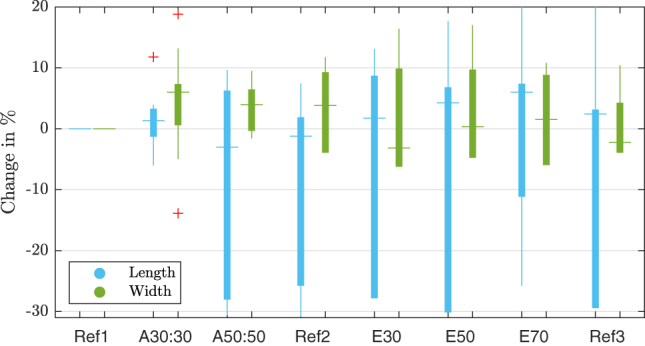
This boxplot shows the change VF of the length in blue and the width in green. The measurements are normalized on the first reference. The boxes indicate the 25th and 75th percentiles, whiskers show the most extreme data points that are included. The horizontal bar in the box indicates the median, excluded outliers are marked with a red ‘+’.

The position of the marker points gives insight how the manipulation effects the VF. In Figure 7 the change of the tissue normalized to the initial measurement can be seen. For the adduction it seems that the VF gets 3.0 % shorter but the lateral surface strain increases about 6.0 %. The following elongation thins the VFs width about 3.2 % and length increases due to the stretching of 6.0 % of the VF.

#### 3.2.2 Effect of manipulations on the fundamental frequency

**Figure 8:**
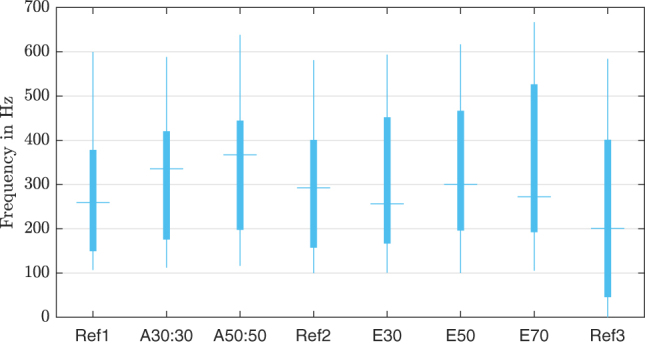
Effect of manipulations on the fundamental frequency *f*_0_.

In Figure 8 the fundamental frequencies can be seen. The frequency starts initially for the first reference measurement Ref1 at a median value of 258.9 Hz. The manipulations do change the frequency. It can be stated, that the obtained adduction levels do show a higher effect on the frequency and do also lead to higher “frequencies” 366.9 Hz compared to the applied elongation where the highest frequency was measured to 300.3 Hz at an elongation with 50 g. The frequency then falls to 200.8 Hz for the last reference measurement.

#### 3.2.3 Glottal resistance

**Figure 9:**
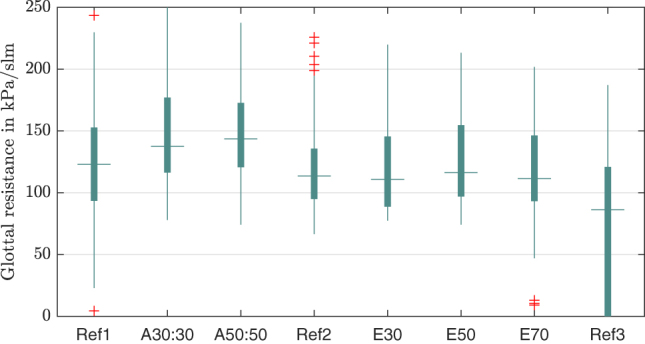
Boxplot of the glottal resistance for each manipulation.

Analogous to the frequency the glottal resistance rises stronger for the adduction. In Figure 9 can be seen that it starts with a median of 123.0 kPa slm^−1^ to increase subsequently to a maximum of 143.5 kPa slm^−1^ with an adduction of 50 g. Applying the elongation the glottal resistance stays around the value of the second reference measurement of 113.6 kPa slm^−1^ and finally drops to 86.2 kPa slm^−1^ for the last unmanipulated reference measurement.

#### 3.2.4 Closing velocity

**Figure 10:**
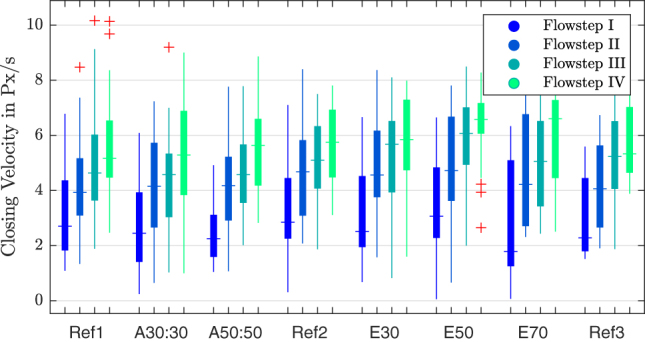
Boxplot of the max. closing velocity for different manipulations, grouped by the flow steps.

In Figure 10 the velocity of the VF for each manipulation can be seen. The values do show a strong trend for different flow rates, but do not react so strongly due to the manipulations. To demonstrate this, the flow rates have been illustrated separately. But one can see that the glottal velocity shows lower values for the adduction and then slightly higher values for the elongation.

#### 3.2.5 Contact pressures

**Figure 11:**
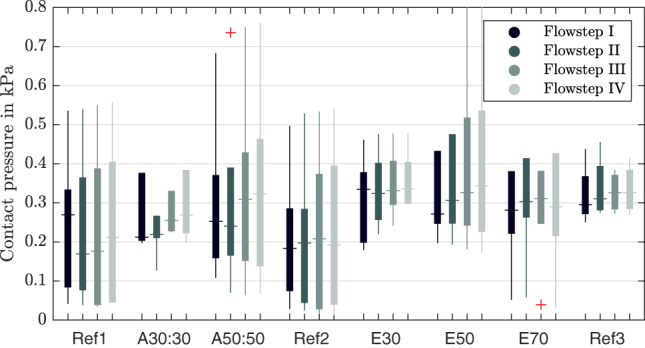
Boxplot of the maximum contact pressures for different manipulations, grouped by the flow steps.

The contact pressure at 50 % of the glottal midline is evaluated and the maximum values are plotted for all manipulations, flow rates and larynges in Figure 11. It can be seen, that with some exceptions, a higher flow rate results in a higher contact pressure in general. The highest median value of 0.34 kPa is reached with an elongation of 50 g. Further it is to notice that the last reference value does not fall back to the initial values of the two other reference measurements.

### 3.3 Correlation between different measured parameters

**Figure 12:**
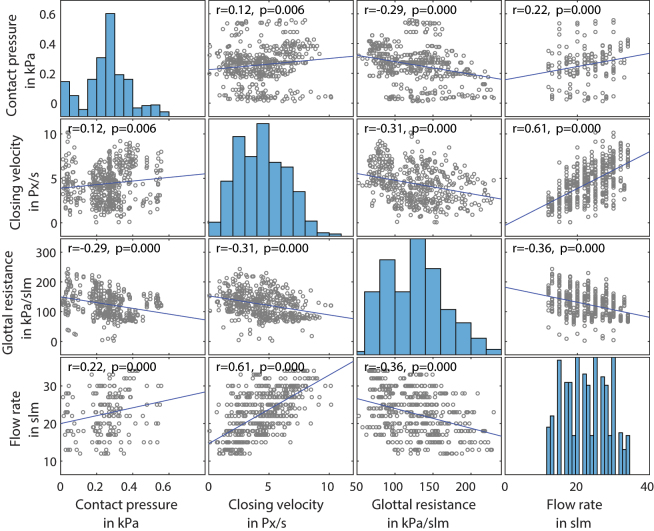
The four parameters contact pressure, closing velocity, glottal resistance and flow rate are plotted against each other and a Pearson-correlation among each other is investigated. The *p* value to neglect the null hypothesis was set to *p* = 0.05.

A data set of seven larynges, with eight manipulations and four flowsteps could be collected, which results in 224 single measurements. This made a statistical evaluation to find correlations underneath different parameters possible. A Pearson-correlation was applied on the data set. As one can see in Figure 12, the contact pressure correlates weakly (0.3 < *r* < 0.5) with the closing velocity as well as with the flow rate, and it shows a negative correlation with the glottal resistance (*r* = −0.29). Furthermore it can be seen that the closing velocity correlates strongly with the flowrate (*r* > 0.61). The extensive experimental setup delivers even more parameters; only the most significant are chosen for demonstrations and those that do not show correlations, were not plotted.

## 4 Discussion

The measurement of the contact pressure between VFs while phonation, even if it is obtained ex vivo, is challenged by the size of the VFs of about 10 mm^2^–20 mm^2^, the small contact pressures between 0.5 kPa and 87.6 kPa and their high oscillation frequency of 100 Hz–300 Hz [11, 20]. Due to the thin and flexible dimension of the film sensor it was possible to include it into the hemi-larynx phonation experiments and gather a data-set of 224 single measurements. The developed read-out unit was able to measure the small acting contact pressures on an area of 15.2 × 30.4 mm^2^ with a high sample rate of 1.2 kfps. Investigating the phonation of seven larynges under eight manipulations with four different flow rates, we could come to the following findings:

**Stretch:** As seen in Figure 7, the manipulations do affect the VF dimensions. By applying the adductions it gets shorter but thicker, and then it is stretched and gets thinner when elongated.

**Frequency:** The obtained adductions show a higher effect on the frequency and do also lead to higher frequencies compared to the applied elongation, which is against the expectation that mainly the elongation pitches the frequency, it can be seen that both manipulations are increasing the frequency and the adduction even more so. In literature an increase of frequency while the VFs are adducted is described by a rise of the sub-glottal pressure. Further it is stated that an increase of 1 kPa rises the phonation about 20 Hz–40 Hz [11]. When regarding the dimension change of the VF in Figure 7 one can see that an elongation even produced an overstretching of the tissue and the third reference measurement Ref3 does not fall back to the initial value. This points to an hysteresis of the tissue.

**Glottal resistance:** Also, the glottal resistance shows higher values for the adduction, in comparison to the elongation.

**Contact pressure:** Compared to other *in vivo* investigation, like Hess et al. [27], or Verdolini et al. [28] who measured values between 1 kPa and 87.6 kPa we measured quite low contact pressures. This may be also a result of the hemi-larynx setup, in literature maximum contact-pressure values of 1.96 kPa were measured by Jiang & Titze [15] during hemi-larynx experiments. To assure the airflow through the glottis and prevent any loss underneath, the larynx had to be mounted quite close to the plate. So, compared to full-larynx experiments the larynx can not move so freely to the manipulations.

**Closing velocity:** The contact pressure results by a deceleration of the vocal fold and therefore results out of the kinetic momentum of the VF. So to say the velocity and the mass of the VF should relate to the contact pressure. By looking at the correlation in Figure 12, one can also see a correlation between those parameters.

**Glottal resistance:** A negative correlation between the glottal resistance and the contact pressure could be found.

It could be clearly shown that a high flow rate results in high contact pressures. This underlines the already known fact, that intense and loud speaking over a longer time can lead to speech problems as hoarseness, which could be traced back to higher contact pressures between the VFs [20]. To come back to our initial research question which was, what type of phonation results in high contact pressures. Beside of loud speaking, through high flow rates, we could show that the glottal resistance correlates negatively to the contact pressure. This could lead to the conclusion that a phonation towards a “pressed” voice tends to have lower contact pressure and a phonation towards a “breathy” one higher pressures. This may sound counterintuitive, but it has to be admitted that the phonations types produced by the manipulations in this study do not represent the whole spectrum of phonation. They are rather an excerpt between the endpoints marked by a “breathy” or “pressed” voice. So, what we can see in the data is, that a phonation during adduction, leads to a higher glottal resistance as can be seen in Figure 9 and to a lower contact pressure compared to an elongation. This might be traced back to the reduced distance between the VF, due to the adduction, resulting in a smaller vibration amplitude. So, the VF cannot reach high closing velocities, which based on the law of impulse conservation lead to lower contact pressures [11].

In future studies it would be interesting to produce more different types of phonation to investigate the effect on the contact pressure in more detail. Furthermore the development of a faster and more sensitive measurement setup would be desirable, too.

## References

[1] Ramig L. O., Verdolini K. (1998). Treatment efficacy: voice disorders. *J. Speech Lang. Hear. Res.*.

[2] Titze I. R., Martin D. W. (1998). Principles of voice production. *J. Acoust. Soc. Am.*.

[3] Gunter H. E., Howe R. D., Zeitels S. M., Kobler J. B., Hillman R. E. (2005). Measurement of vocal fold collision forces during phonation. *J. Speech Lang. Hear. Res.*.

[4] Huang Y.-P., Zheng Y.-P., Wang S.-Z., Chen Z.-P., Huang Q.-H., He Y.-H. (2009). An optical coherence tomography based air jet indentation system for measuring the mechanical properties of soft tissues. *Meas. Sci. Technol.*.

[5] Lamprecht R., Scheible F., Semmler M., Sutor A. (2021). A quasi-static quantitative ultrasound elastography algorithm using optical flow. *Sensors*.

[6] Lamprecht R., Scheible F., Veltrup R. (2022). Quasi-static ultrasound elastography of ex-vivo porcine vocal folds during passive elongation and adduction. *J. Voice*.

[7] Hsiao T.-Y., Wang C.-L., Chen C.-N., Hsieh F.-J., Shau Y.-W. (2002). Elasticity of human vocal folds measured in vivo using color Doppler imaging. *Ultrasound Med. Biol.*.

[8] Kazemirad S., Bakhshaee H., Mongeau L., Kost K. (2014). Non-invasive in vivo measurement of the shear modulus of human vocal fold tissue. *J. Biomech.*.

[9] Sharma G. K., Chen L. Y., Chou L. (2021). Surface kinematic and depth-resolved analysis of human vocal folds in vivo during phonation using optical coherence tomography. *J. Biomed. Opt.*.

[10] Scheible F., Lamprecht R., Semmler M., Sutor A. (2021). Dynamic biomechanical analysis of vocal folds using pipette aspiration technique. *Sensors*.

[11] Behrman A. (2017). *Speech and Voice Science*.

[12] Sundberg J. (1977). *The Acoustics of the Singing Voice*.

[13] Sundberg J. (1988). *The Science of the Singing Voice*.

[14] Motie-Shirazi M., Zañartu M., Peterson S. D. (2019). Toward development of a vocal fold contact pressure probe: sensor characterization and validation using synthetic vocal fold models. *Appl. Sci.*.

[15] Jiang J. J., Titze I. R. (1994). Measurement of vocal fold intraglottal pressure and impact stress. *J. Voice*.

[16] Díaz-Cádiz M. E., Peterson S. D., Galindo G. E. (2019). Estimating vocal fold contact pressure from raw laryngeal high-speed videoendoscopy using a hertz contact model. *Appl. Sci.*.

[17] Verdolini K., Chan R., Titze I. R., Hess M., Bierhals W. (1998). Correspondence of electroglottographic closed quotient to vocal fold impact stress in excised canine larynges. *J. Voice*.

[18] Tekscan Pressure-mapping-sensor 4201 datasheet. ..

[19] Scheible F., Veltrup R., Schaan C. (2021). Measuring contact pressures of phonating vocal fold using a pressure-sensing-matrix during hemi-larynx experiments. *Proceedings of Meetings on Acoustics*.

[20] Miri A. K. (2014). Mechanical characterization of vocal fold tissue: a review study. *J. Voice*.

[21] Döllinger M., Berry D. A., Kniesburges S. (2016). Dynamic vocal fold parameters with changing adduction in ex-vivo hemilarynx experiments. *J. Acoust. Soc. Am.*.

[22] Scheible F., Lamprecht R., Schaan C. (2023). Behind the complex interplay of phonation: investigating elasticity of vocal folds with pipette aspiration technique during ex vivo phonation experiments. *J. Voice*.

[23] Kist A. M., Gómez P., Dubrovskiy D. (2021). A deep learning enhanced novel software tool for laryngeal dynamics analysis. *J. Speech Lang. Hear. Res.*.

[24] Semmler M., Berry D. A., Schützenberger A., Döllinger M. (2021). Fluid-structure-acoustic interactions in an ex vivo porcine phonation model. *J. Acoust. Soc. Am.*.

[25] van den Berg J., Zantema J. T., Doornenbal P. (1957). On the air resistance and the Bernoulli effect of the human larynx. *J. Acoust. Soc. Am.*.

[26] Zhang Z. (2019). Vocal fold contact pressure in a three-dimensional body-cover phonation model. *J. Acoust. Soc. Am.*.

[27] Hess M. M., Verdolini K., Bierhals W., Mansmann U., Gross M. (1998). Endolaryngeal contact pressures. *J. Voice*.

[28] Verdolini K., Hess M. M., Titze I. R., Bierhals W., Gross M. (1999). Investigation of vocal fold impact stress in human subjects. *J. Voice*.

